# PQM130, a Novel Feruloyl–Donepezil Hybrid Compound, Effectively Ameliorates the Cognitive Impairments and Pathology in a Mouse Model of Alzheimer’s Disease

**DOI:** 10.3389/fphar.2019.00658

**Published:** 2019-06-12

**Authors:** Fabiana Morroni, Giulia Sita, Agnese Graziosi, Gloria Ravegnini, Raffaella Molteni, Maria Serena Paladini, Kris Simone Tranches Dias, Ariele Faria dos Santos, Claudio Viegas, Ihosvany Camps, Letizia Pruccoli, Andrea Tarozzi, Patrizia Hrelia

**Affiliations:** ^1^Department of Pharmacy and BioTechnology–FaBiT, Alma Mater Studiorum–University of Bologna, Bologna, Italy; ^2^Department of Medical Biotechnology and Translational Medicine, University of Milan, Milan, Italy; ^3^Institute of Chemistry, Federal University of Alfenas, Alfenas, MG, Brazil; ^4^Institute of Exact Sciences, Federal University of Alfenas, Alfenas, MG, Brazil; ^5^Department for Life Quality Studies-QuVi, Alma Mater Studiorum-University of Bologna, Rimini, Italy

**Keywords:** Alzheimer’s disease, amyloid-β oligomers, oxidative stress, apoptosis, neuroprotection, multitarget ligand, drug discovery, feruloyl-donepezil hybrid

## Abstract

Alzheimer’s disease (AD) is the most frequent type of dementia in older people. The complex nature of AD calls for the development of multitarget agents addressing key pathogenic processes. Donepezil, an acetylcholinesterase inhibitor, is a first-line acetylcholinesterase inhibitor used for the treatment of AD. Although several studies have demonstrated the symptomatic efficacy of donepezil treatment in AD patients, the possible effects of donepezil on the AD process are not yet known. In this study, a novel feruloyl–donepezil hybrid compound (PQM130) was synthesized and evaluated as a multitarget drug candidate against the neurotoxicity induced by Aβ_1-42_ oligomer (AβO) injection in mice. Interestingly, PQM130 had already shown anti-inflammatory activity in different *in vivo* models and neuroprotective activity in human neuronal cells. The intracerebroventricular (i.c.v.) injection of AβO in mice caused the increase of memory impairment, oxidative stress, neurodegeneration, and neuroinflammation. Instead, PQM130 (0.5–1 mg/kg) treatment after the i.c.v. AβO injection reduced oxidative damage and neuroinflammation and induced cell survival and protein synthesis through the modulation of glycogen synthase kinase 3β (GSK3β) and extracellular signal–regulated kinases (ERK1/2). Moreover, PQM130 increased brain plasticity and protected mice against the decline in spatial cognition. Even more interesting is that PQM130 modulated different pathways compared to donepezil, and it is much more effective in counteracting AβO damage. Therefore, our findings highlighted that PQM130 is a potent multi-functional agent against AD and could act as a promising neuroprotective compound for anti-AD drug development.

## Introduction

The World Health Organization estimated the presence of 47.5 million people worldwide with dementia in 2015 and predicted that the number of patients will be almost tripled by 2050 (https://www.who.int/mental_health/neurology/dementia/en/). Mainly owing to significant increases in lifespan, dementia represents one of the major global health crises of the 21st century. The most widespread form of dementia is Alzheimer’s disease (AD). AD is a lethal neurodegenerative illness that begins with brain alterations more than 20 years before the clinical symptoms (Mori et al., 2019). This multifaceted and progressive neurodegenerative disease is pathologically characterized by the amyloid-β (Aβ) accumulation in amyloid plaques and the hyperphosphorylation of tau in neurofibrillary tangles, followed by a consistent neuronal loss leading to brain atrophy and dementia. Although scientific research has changed course from fibrillar Aβ, implicated in plaque formation, to soluble Aβ, whose accumulation is probably the cause of the early synaptic dysfunction (Selkoe, 2002), the protein is still considered the keystone of AD. Levels of soluble Aβ oligomers (AβO) have been shown in several experimental models to potently inhibit hippocampal long-term potentiation (LTP), increase dendritic spine loss, and impair cognition in mice (Walsh et al., 2002; Lacor et al., 2007; Morroni et al., 2016; Herline et al., 2018). Although AD progression is tightly connected to Aβ aggregation, the scientific consensus is quite firm in suggesting that several other factors likely contribute to the development of AD. Such factors include loss of cholinergic transmission, mitochondrial dysfunction, progressive oxidative damage, excitotoxicity, and neuroinflammatory processes, which may trigger a “domino” cascade of events leading to manifestation of AD (Macchi et al., 2014; Hampel et al., 2018; Pérez et al., 2018). It is likely that AD begins as a synaptic disorder and decreased synaptic activity is one of the best pathological signal of cognitive decline in AD (Coleman and Yao, 2003). Brain-derived neurotrophic factor (BDNF) is a pleiotropic growth factor in the brain, and it plays a crucial role in the survival and neuronal function (Hu et al., 2019). Indeed, not only can it modulate synapse formation and neurogenesis, but it can also reduce oxidative stress and cell death. In the early stage of AD, the levels of the precursor form of BDNF, mature BDNF, or its mRNA are reduced in the parietal cortex and hippocampus (Phillips et al., 1991; Peng et al., 2005; Song et al., 2015).

There is currently no cure for AD. Unfortunately, the AD clinical trials targeting Aβ to date have been unsuccessful, demonstrating the need to investigate innovative therapeutic approaches beyond Aβ, and trying to focus attention on other early key events, in particular synaptic dysfunction, oxidative stress, or the early events of neuroinflammation (Marttinen et al., 2018). Thus, it is likely reasonable to argue that multifactorial diseases, such as AD, cannot be successfully treated by modulating a single target, but they will require multitarget drug treatment to address the different pathological facets of these diseases.

Acetylcholinesterase (AChE) inhibitors and *N*-methyl-d-aspartate antagonists are the current therapies for AD-related symptoms with poor efficacy and no evidence of disease modification (Lanctôt et al., 2009). Donepezil is a highly centrally selective, reversible, and non-competitive AChE inhibitor and currently the most frequently prescribed drug for the treatment of AD. Clinical trials with donepezil have highlighted slight but reproducible improvements in cognitive function of the treated patients as compared to placebo. However, these effects were transient because cognitive function continued to decline over time in patients (Doody et al., 2007).

**Figure 1:**
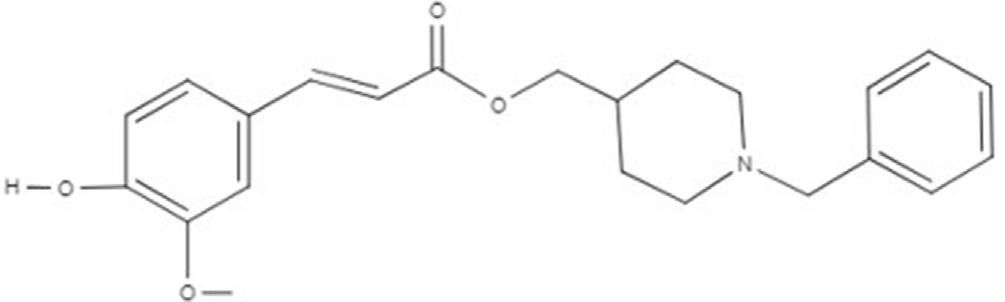
Chemical structure of PQM130.

As a consequence of the failure of one target–one ligand approach to provide promising results in AD treatment, new findings suggested that one molecule hitting multiple targets could represent the winning strategy to treat complex diseases (Schmitt et al., 2004). Thus, “the multi-target-directed ligand (MTDL) approach is based on the design of new scaffolds with different pharmacophoric subunits connected in a single molecule, which could modulate multiple molecular targets at the same time” (Dias et al., 2017). Considering the MTDL approach, we studied here the activity of the multitarget ligand PQM130 (**Figure 1**), which is the most promising compound of a new series of molecular hybrids synthesized by the combination of two subunits, the *N*-benzylpiperidine group present in donepezil and responsible for its AchE selectivity, linked to the feruloyl group present in ferulic acid (Dias et al., 2017). Ferulic acid is one of the degradation products of curcumin, which has already shown neuroprotective activities probably due to its ability to modify the kinetics of Aβ fibril formation, as well as to its anti-oxidative and anti-inflammatory activities (Hamaguchi et al., 2010; Sgarbossa et al., 2015). The multitarget ligand PQM130 has already been investigated for its *in vitro* anticholinesterase, metal-chelating, antioxidant, neuroprotective, and anti-inflammatory properties, in different *in vivo* models (Dias et al., 2017). Moreover, PQM130 also highlighted an interesting pharmacokinetic profile from the *in silico* evaluation of the absorption, distribution, metabolism, elimination (ADME) parameters, using the software QikProp 3.1 (Schrödinger, LLC, New York, NY, USA; see **Supplementary Material 1**) (Dias Viegas et al., 2018). Interestingly, ADME data of PQM130 showed a good human absorption and blood–brain barrier penetration in accordance with the software reference parameters (Dias Viegas et al., 2018). A similar *in silico* approach was adopted to evaluate the PQM130 safety, using the VEGA platform (https://www.vegahub.eu/; Mario Negri Institute for Pharmacological Research, Milan, Italy), which includes various QSAR models. In particular, mutagenicity (CONSENSUS) and carcinogenicity (IRFMN/ANTARES) models reported the absence of mutagenic and carcinogen effects of PQM130 (see **Supplementary Material 2** and **3**).

In the current study, we have further examined the neuroprotective effects of the multitarget ligand PQM130 in comparison also to donepezil in a mouse AD model generated by intracerebroventricular (i.c.v.) injection of Aβ_1-42_ oligomers (Aβ_1-42_O) and discussed the molecular mechanisms with particular attention to its nootropic, neuroprotective, and neurotrophic activities.

## Materials and Methods

### Reagents

Aβ_1–42_ peptides were purchased by AnaSpec (Fremont, CA, USA). Aprotinin, bovine serum albumin (BSA), CHAPS, 2’7’-dichlorodihydrofluorescein diacetate (DCFH-DA), dimethyl sulfoxide, 5,5′-dithiobis (2-nitrobenzoic acid), dithiothreitol, donepezil hydrochloride, EDTA, eosin, ethanol, glycerol, hematoxylin, Hepes pH 7.4, hexafluoroisopropanol, leupeptin, β-mercaptoethanol, sodium chloride, sodium fluoride, sodium orthovanadate, sucrose, sulfosalicylic acid, Triton-X 100, tris pH 7.5, xylen, and primary antibodies anti-synaptophysin and anti-β-actin were provided by Sigma-Aldrich (St Louis, MO, USA). Paraformaldehyde solution (4%) was provided by Santa Cruz Biotechnology (Dallas, TX, USA) and NP-40 was from Roche Diagnostic (Risch, Switzerland). Caspase substrates were purchased from Alexis Biochemicals (San Diego, CA, USA). Primary antibodies phospho-GSK3α/β (Ser21/9) and GSK3α/β, phospho-p44/42 MAPK (ERK1/2, Thr202/Tyr204) and p44/42 MAPK, and anti-GFAP were provided by Cell Signaling Technologies Inc. (Danvers, MA, USA). Secondary anti-mouse and anti-rabbit antibodies were purchased from GE Healthcare (Piscataway, NJ, USA) and fluorescein was from Life Technologies (Carlsbad, CA, USA). Bradford assay solution, enhanced chemiluminescence (ECL) solution, Tris-buffered saline (TBS), and Tween 20 were purchased from Bio-Rad Laboratories S.r.L. (Hercules, CA, USA). Normal goat serum (NGS) was provided by Wako Pure Chemical Industries (Osaka, Japan). All experiment reagents were reagent grade and commercially available.

### Animals

Adult male C57Bl/6 mice (9 weeks old, 25–30 g body weight; Harlan, Milan, Italy) were utilized. The mice were housed in a temperature-controlled room (23–24°C) with free access to food and water and presented with 12 h light/12 h dark cycles. Briefly, procedures on the mice were carried out according to the European Communities Council Directive 2010/63/EU and the current Italian Law on the welfare of the laboratory animal (D.Lgs. n.26/2014). The animal protocol was approved by the Italian Ministry of Health (Authorization No. 291/2017-PR) and by the corresponding committee at the University of Bologna. The number of experimental animals was minimized and care was taken to limit mice suffering.

#### Experimental Design

**Figure 2:**
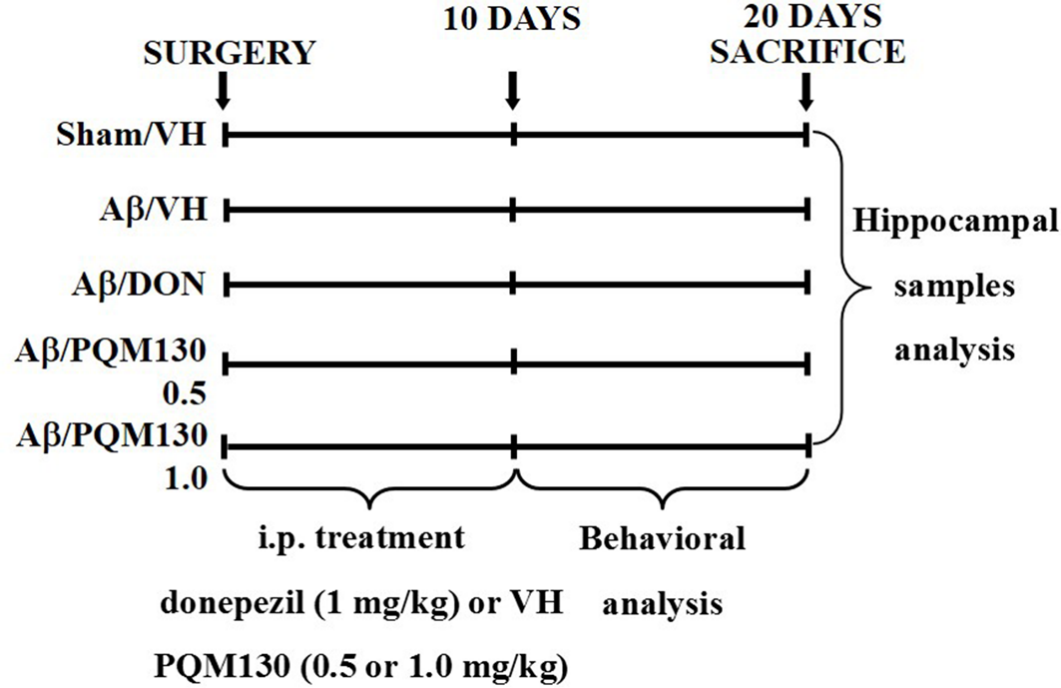
Experimental protocol and treatment schedule. The mice received i.p. injections of DON (1 mg/kg) or PQM130 (0.5 or 1.0 mg/kg) or VH solution for 10 days. The animals were sacrificed 20 days after Aβ_1-42_ oligomer injection.

The animals were randomized into five groups (*n* = 10/group): Sham/VH, Aβ/VH, Aβ/DON, Aβ/PQM130 0.5 mg/kg, and Aβ/PQM130 1.0 mg/kg. Four groups were treated with Aβ_1-42_O by a unilateral i.c.v. injection, while the other received a unilateral i.c.v. injection of saline solution (sham group). One hour after the brain lesion, mice received intraperitoneal (i.p.) treatment of 1 mg/kg of donepezil hydrochloride (DON, Sigma-Aldrich), 0.5 or 1 mg/kg of PQM130, or vehicle (VH, saline). The dose injected was selected according to the literature (Furukawa-Hibi et al., 2011; Dias et al., 2017). We treated the mice daily for 10 days. At the conclusion of the treatment period, the mice underwent behavioral assessment. After the behavioral analysis, the animals were deeply anesthetized before being sacrificed by cervical dislocation to collect the samples for immunohistochemical and neurochemical analysis (for experimental design, see **Figure 2**).

#### A**β**_1-42_ Oligomers Preparation and Injection

Aβ_1–42_ peptides (AnaSpec) were solubilized to 1 mg/ml in hexafluoroisopropanol before being sonicated and lyophilized at room temperature. The unaggregated Aβ_1–42_ film obtained was dissolved to a final concentration of 1 mM with sterile dimethyl sulfoxide and stored at −20°C until use. The Aβ_1–42_O were prepared according to the protocol of Tarozzi et al. (2008). Briefly, to enhance oligomer formation, the Aβ_1-42_ stock was diluted in saline buffer at 40 μM and incubated for 48 h at 4°C (Hong et al., 2007; Maezawa et al., 2008). Six microliters of Aβ_1-42_O (40 μM) were injected i.c.v., using a stereotaxic mouse frame (myNeuroLab, Leica-Microsystems Co., St. Louis, MO, USA) and a 10-µL Hamilton syringe, at a rate of 0.5 ml/min. After the injection, the needle was left in place for a few minutes before being retracted slowly and the wound was cleaned and sutured. The sham mice received the corresponding volume of saline. The following coordinates were used: anteroposterior: +0.22, mediolateral: +1.0, dorsoventral: −2.5, with a flat skull position.

#### Donepezil Hydrochloride and PQM130 Preparations

Donepezil hydrochloride was purchased from Sigma-Aldrich and PQM130 (purity 98% by HPLC) was synthesized and provided by Professor Claudio Viegas Jr from the PeQuiM-Laboratory of Research in Medicinal Chemistry, Institute of Chemistry, Federal University of Alfenas (Alfenas, MG, Brazil). Briefly, the powders were solubilized and aliquoted in sterilized saline (donepezil) or in dimethyl sulfoxide (PQM130). The work solutions were prepared at a concentration of 0.1 mg/ml (donepezil and PQM130) and 0.05 mg/ml (PQM130) in sterilized saline. Animals were daily i.p. injected with 1 mg/kg solution (donepezil and PQM130) or 0.5 mg/kg (PQM130) for 10 days.

### Behavioral Analysis

All the tests were performed between 9.30 a.m. and 3.30 p.m. All scores were attributed by a blinded observer.

#### Morris Water Maze (MWM)

The test was performed as described previously (Morroni et al., 2018a). Briefly, the apparatus was a circular plastic tank (1.0 m diameter, 50 cm height) filled with water and milk (22°C), and a submerged platform (1.5 cm under the water surface) positioned in the center of one of the four quadrants of the maze. A camera was placed to register mice’s movements and send data to an automated tracking system (EthoVision, Noldus, The Netherlands). For each training trial, animals were placed into the pool at one of the four positions selected randomly, and the latency to find the hidden platform was recorded. Mice that could not reach the platform within 60 s were guided to it by the experimenter. After the trial, each mouse was placed under a warming lamp in a holding cage for 25 s until the next trial. Training trials were conducted four times a day for 5 days. On day 6, the platform was removed and animals were allowed to swim freely for 60 s. The parameters measured during the probe trial were escape latency, frequency in the platform zone, and time spent in the opposite quadrant to the platform zone.

#### Y-Maze Test

The spatial working memory was evaluated by recording spontaneous alternation behavior in the Y-maze as described earlier (Sarter et al., 1988). Briefly, each arm of the maze [Ugo Basile^®^ S.r.L., Gemonio (VA), Italy] was 35 cm long, 15 cm high, and 5 cm wide and converged to a 120° angle. The mice were positioned at the end of the A arm and allowed to move freely through the maze for 5 min. The entry in all three arms consecutively was counted as an alternation. Thus, the number of maximum alternations was calculated as the total number of arm entries minus two and the percentage of alternation was calculated as (actual alternations/maximum alternations) × 100 (Lopes et al., 2018).

### Tissue Preparation for Immunohistochemistry and Neurochemical Analysis

At the end of behavioral tests, the mice were deeply anesthetized and sacrificed by cervical dislocation. The brains were quickly removed and one hemisphere of each mouse was fixed in 4% paraformaldehyde (Santa Cruz Biotechnology) for 48 h. The other hemispheres were immediately removed, and the hippocampi were isolated on ice and transferred to liquid nitrogen.

For the protein extraction, the tissues were homogenized in lysis buffer and the cytoplasmic protein concentration was determined by the Bradford method (Bradford, 1976).

#### Determination of Caspase-9 and -3 Activations

Caspase-9 and -3 enzyme activities were measured according to Movsesyan et al. (2002). Briefly, the tissue lysates were incubated with the assay buffer and a 50 mmol/L concentration of chromogenic *p*-nitroaniline (pNA) substrate (caspase-9, Ac-Leu-Glu-His-Asp-pNA; caspase-3, Z-Asp-Glu-Val-Asp-pNa; Alexis Biochemicals). Each sample was incubated for 3 h at 37°C and the amount of pNA released was measured with a microplate reader (GENios, TECAN^®^, Mannedorf, Switzerland) at 405 nm. The values were expressed as the mean ± SEM of optical density (OD) of each experimental group.

#### Determination of Cellular Redox Status

The redox status, in terms of reactive oxygen species (ROS) formation, was evaluated by measuring the oxidation of DCFH-DA to 2′7′-dichlorofluorescein (DCF) (Morroni et al., 2014). The samples (60 μl) were incubated for 30 min with 2 mg/ml of DCFH-DA, and the conversion into the fluorescent product DCF was measured (excitation at 485 nm, emission at 535 nm) using a microplate reader (GENios, TECAN^®^). The values were normalized to protein content and expressed as the mean ± SEM of fluorescence intensity arbitrary units (UF) of each experimental group.

#### Determination of Glutathione Content

Glutathione (GSH) content was assessed using the protocol described earlier (Morroni et al., 2018b). Briefly, samples were deproteinized with 4% sulfosalicylic acid, and the supernatants were added to 5,5′-dithiobis (2-nitrobenzoic acid) (4 mg/ml). The developed coloration was read quickly at 412 nm (GENios, TECAN^®^) and the results were calculated using a standard calibration curve. The values were normalized to protein content and expressed as the mean ±SEM of GSH mmol/mg protein of each experimental group.

### Western Blotting

The samples (30 μg proteins) were run on 4–15% SDS polyacrylamide gels (Bio-Rad Laboratories S.r.L.) and electroblotted onto 0.45 μm nitrocellulose membranes. The membranes were incubated at 4°C overnight with primary antibody recognizing phospho-GSK3α/β (Ser21/9), phospho-p44/42 MAPK (ERK1/2, Thr202/Tyr204) (1:1,000; Cell Signaling Technology Inc), or anti-synaptophysin (1:1,000; Sigma-Aldrich). After washing with TBS-T (TBS + 0.05% Tween20), the membranes were incubated with secondary antibodies (1:2,000; GE Healthcare). ECL was used to visualize the bands (Bio-Rad Laboratories). The membranes were then reprobed with GSK3α/β, p44-42 MAPK (1:1,000; Cell Signaling Technology Inc.), or anti-β-actin (1:1,000; Sigma-Aldrich). The data were analyzed by densitometry, using Quantity One software (Bio-Rad Laboratories^®^ S.r.L.). The values were normalized and expressed as the mean ± SEM of the densitometry in each experimental group.

### Immunohistochemistry

The fixed brains were sliced on a vibratome (Leica Microsystems, Milan, Italy) at 40 μm thickness, and the slices were stained as described earlier (Morroni et al., 2016).

#### Hematoxylin/Eosin Staining

Hematoxylin/eosin (H&E) staining was assessed as previously illustrated (Fischer et al., 2008). Briefly, the selected sections were rehydrated by a graded series of alcohols (Sigma-Aldrich). Then, the slices were counterstained in hematoxylin for 8 min and then rinsed for 10 min in tap water. Subsequently, the slices were immersed in distilled water and then in 80% ethanol before being stained in 25% eosin solution (in ethanol 80%) for 1 min. Finally, the slices were dehydrated with graded alcohol before being fixed in xylen.

#### Anti-Glial Fibrillary Acidic Protein (GFAP) Staining

The immunofluorescence staining was assessed according to our previous study (Morroni et al., 2018a). Selected slices were rinsed in phosphate buffer and then incubated in TBS-A (TBS 0.1% Triton-X 100) and TBS-B (TBS-A 2% BSA) to reduce a specific absorption. The sections were then incubated with anti-GFAP primary antibody (1:300; Cell Signaling Technology Inc.) in TBS-B with 3% NGS (Wako Pure Chemical Industries) at 4°C overnight. After 24 h, the slices were washed with TBS-A and TBS-B before being incubated with secondary antibody (1:200; Fluorescein, Life Technologies) in TBS-B with 3% NGS. To verify the binding specificity, some sections were incubated with only primary or secondary antibody. In these conditions, we did not find any positive staining.

#### Quantitative Images Analysis

Image analysis was conducted by an investigator unaware of the treatment groups, using a microscope (AxioImager M1, Carl Zeiss, Oberkochen, Germany) and an image analysis system (AxioCam MRc5, Carl Zeiss) equipped with dedicated software (AxioVision Rel 4.8, Carl Zeiss). The hippocampal region was defined at low magnification (2.5× objective), and the H&E or GFAP staining was evaluated by densitometry of five different sections for each sample analyzed at a higher magnification (10×, 20×, or 40× objective). Quantification and morphological analysis were assessed with the ImageJ software.

### RNA Preparation and Gene Expression Analysis

Total RNA was isolated from hippocampus using the Pure link RNA mini kit (Ambion, Thermo Fisher Scientific, Carlsbad, CA, USA), as illustrated earlier (Morroni et al., 2018b). Briefly, the samples were lysed on ice with 1% β-mercaptoethanol by using a homogenizer SHM1 (Stuart, Bibby Scientific LTD, Staffordshire, UK). The samples were then added to an equal volume of 70% ethanol. The solution was filtered using a cartridge containing a clear silica-based membrane to which the RNA binds. RNA was finally eluted with RNase-free water and stored at −80°C. RNA was quantified by spectrophotometric analysis and reverse-transcribed using High Capacity cDNA Reverse Transcription kit (Applied Biosystems, Thermo Fisher Scientific).

The mRNA encoding for the mouse nuclear factor (erythroid-derived 2)-like 2 (Nrf2), GSH reductase (GR), tumor protein 53 (TP53), and the actin (ACTB) as internal reference were quantified by Taqman RT-PCR with a 7900HT Fast Real-Time PCR system (Applied Biosystems). The samples were run in 96-well format in triplicate. The specific Taqman gene expression assays (Applied Biosystems) were Nrf2 (Mm0047784_m1), GSTP1 (Mm04213618_gH), GR (Mm00439154_m1), TP53 (Mm01731290_g1), and ACTB (Mm00607939_s1).

To assess mRNA levels of different BDNF transcripts (total form, long 3′UTR form, exon IV, exon VI) and synaptophysin, samples were processed for RT-PCR reaction and subsequently analyzed by qRT-PCR instrument (CFX384 Real-Time system, Bio-Rad Laboratories S.r.l.) using the iScript one-step RT-PCR kit for probes (Bio-Rad Laboratories S.r.l.). The samples were run in 384-well format in triplicate as multiplexed reactions with a normalizing internal control (ACTB). The primers and probe sequences, respectively, were as follows: total BDNF (Fwd: AAGTCTGCATTACATTCCTCGA, Rev: GTTTTCTGAAAGAGGGACAGTTTAT, Probe: TGTGGTTTGTTGCCGTTGCCAAG), long 3′UTR BDNF (Fwd: GTTGTCATTGCTTTACTGGCG, Rev: AATTTTCTCCATCCCTACTCCG, Probe: AATCTACCCCTCCCATTCCCCGT), BDNF exon IV (Fwd: AGCTGCCTTGATGTTTACTTTG, Rev: CGTTTACTTCTTTCATGGGCG, Probe: AGGATGGTCATCACTCTTCTCACCTGG), BDNF exon VI (Fwd: GGACCAGAAGCGTGACAAC, Rev: ATGCAACCGAAGTATGAAATAACC, Probe: ACCAGGTGAGAAGAGTGATGACCATCC), Synaptophysin (Fwd: CCTGTCCGATGTGAAGATGG, Rev: AGGTTCAGGAAGCCAAACAC, Probe: ACACATGCAAGGAACTGAGGGACC), and ACTB (Fwd: ACCTTCTACAATGAGCTGCG, Rev: CTGGATGGCTACGTACATGG, Probe: TCTGGGTCATCTTTTCACGGTTGGC).

Each RT-PCR run followed the manufacturer’s conditions: an incubation at 50°C for 10 min (RNA retrotranscription), followed by a step at 95°C for 5 min (TaqMan polymerase activation). Subsequently, 39 cycles of PCR were performed (95°C for 10 s, and then 30 s at 60°C). A comparative cycle threshold (Ct) method was used to determine the relative target gene expression versus the sham group (Rossetti et al., 2016). Specifically, a fold change for each target gene relative to ACTB was determined by the 2^−Δ(ΔCt)^ method, where ΔCt = Ct, target – Ct, β-actin; Δ(ΔCt) = Ct, exp. group – Ct, control group and Ct is the threshold cycle. For graphical clarity, the obtained data were then expressed as percentage versus the Sham/VH, which has been set at 100%.

### Statistical Analysis

The data were analyzed with the PRISM 5 software (GraphPad Software, La Jolla, CA, USA) and expressed as mean ± SEM of each experimental group. The difference between the groups was analyzed by one-way ANOVA with Bonferroni *post hoc* test. The results were considered statistically significant when a *p* value was less than 0.05.

## Results

### PQM130 Ameliorated Aβ_1-42_O-Induced Cognitive Deficits in Mice

**Figure 3:**
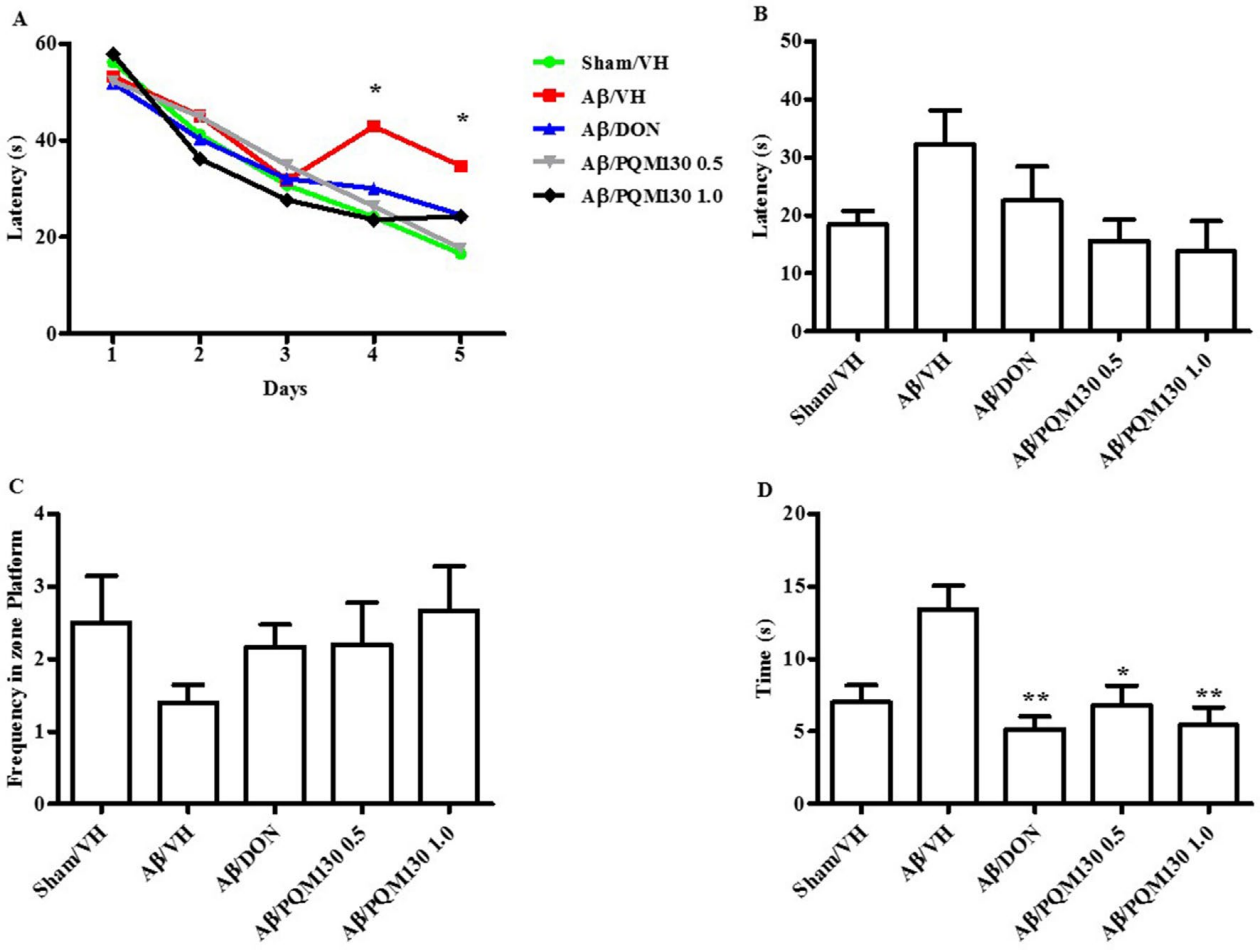
Effects of donepezil and PQM130 treatments (0.5 or 1.0 mg/kg) on the performance in the training **(A)** and probe trials **(B–D)** of the MWM test in the Aβ_1-42_O-injected mice. The training trials were carried out for 5 days (four per day); the probe trial was performed on day 6. The escape latency **(B)**, the frequency in the platform zone **(C)**, and the time spent in the opposite quadrant to the platform zone **(D)** were recorded in the probe test. The values are expressed as mean ± SEM (*n* = 10) (**A**: *p < 0.05 vs. Aβ/VH group; **D**: *p < 0.05 and **p < 0.01 vs. Aβ/VH; ANOVA, *post hoc* test Bonferroni).

**Figure 4:**
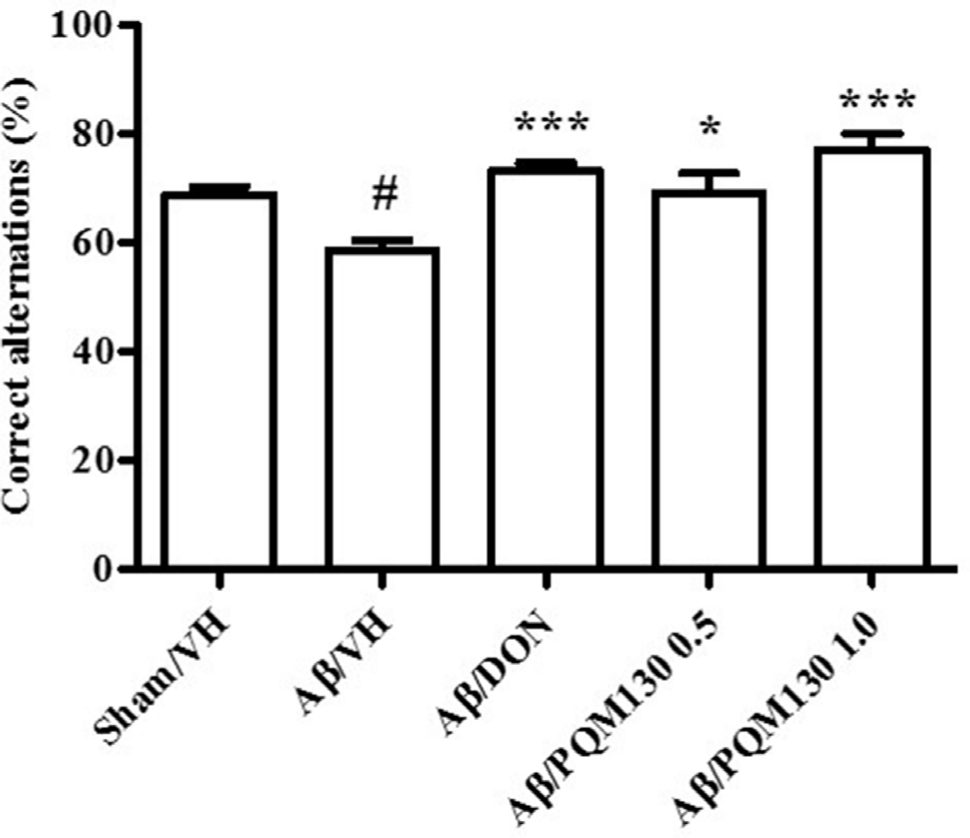
Effects of donepezil and PQM130 treatments (0.5 or 1.0 mg/kg) on the performance in the Y-maze test in the Aβ_1-42_O-injected mice. The spontaneous alternation percentage was recorded in a 5 min trial. The values are expressed as mean ± SEM (*n* = 10) (^#^ *p* < 0.05 vs. Sham/VH, *p < 0.05 and ***p < 0.001 vs. Aβ/VH; ANOVA, *post hoc* test Bonferroni).

The i.c.v. injection of Aβ_1-42_O induced cognitive impairment as shown in the MWM and Y-maze tests. During the MWM training phase, all the mice learned the platform location, as clearly highlighted by the decreased latency and the distance traveled to find the platform. However, the Aβ/VH mice needed more time and traveled a longer distance to locate the platform than the sham mice, which undoubtedly highlighted a short-term memory impairment in these mice. From the fourth day of training, the treated groups (Aβ/DON and Aβ/PQM130) showed a significantly lower escape latency than those in the Aβ/VH group (*p* < 0.05; **Figure 3A**). The swimming speed was not significantly different among the groups during the training (data not shown). In the probe trial, the mice in the Aβ/VH group revealed difficulties in locating the original position of the removed platform (longer latency to first enter the target zone, less frequency crossing the platform, and more time spent swimming in the opposite quadrant; **Figure 3B–D**). Interestingly, the Aβ/DON and Aβ/PQM130 mice performed better than the Aβ/VH, even though significantly only with regard to time spent in the opposite quadrant (donepezil *p* < 0.01; PQM130 *p* < 0.05 and *p* < 0.01, respectively). In the Y-maze test, which assesses spatial working memory, the spontaneous alternation behavior of Aβ/VH group was significantly lower than the sham group (*p* < 0.05, **Figure 4**), confirming the difficulty in remembering which arm has already been visited. This behavioral impairment was significantly improved in the Aβ/DON and Aβ/PQM130 groups (donepezil *p* < 0.001; PQM130 *p* < 0.05 and *p* < 0.001, respectively), demonstrating that DON and PQM130 could effectively increase spatial working memory in the early stage of AD development.

### PQM130 Prevented Aβ_1-42_O-Induced Neuronal Death in Mice

**Figure 5:**
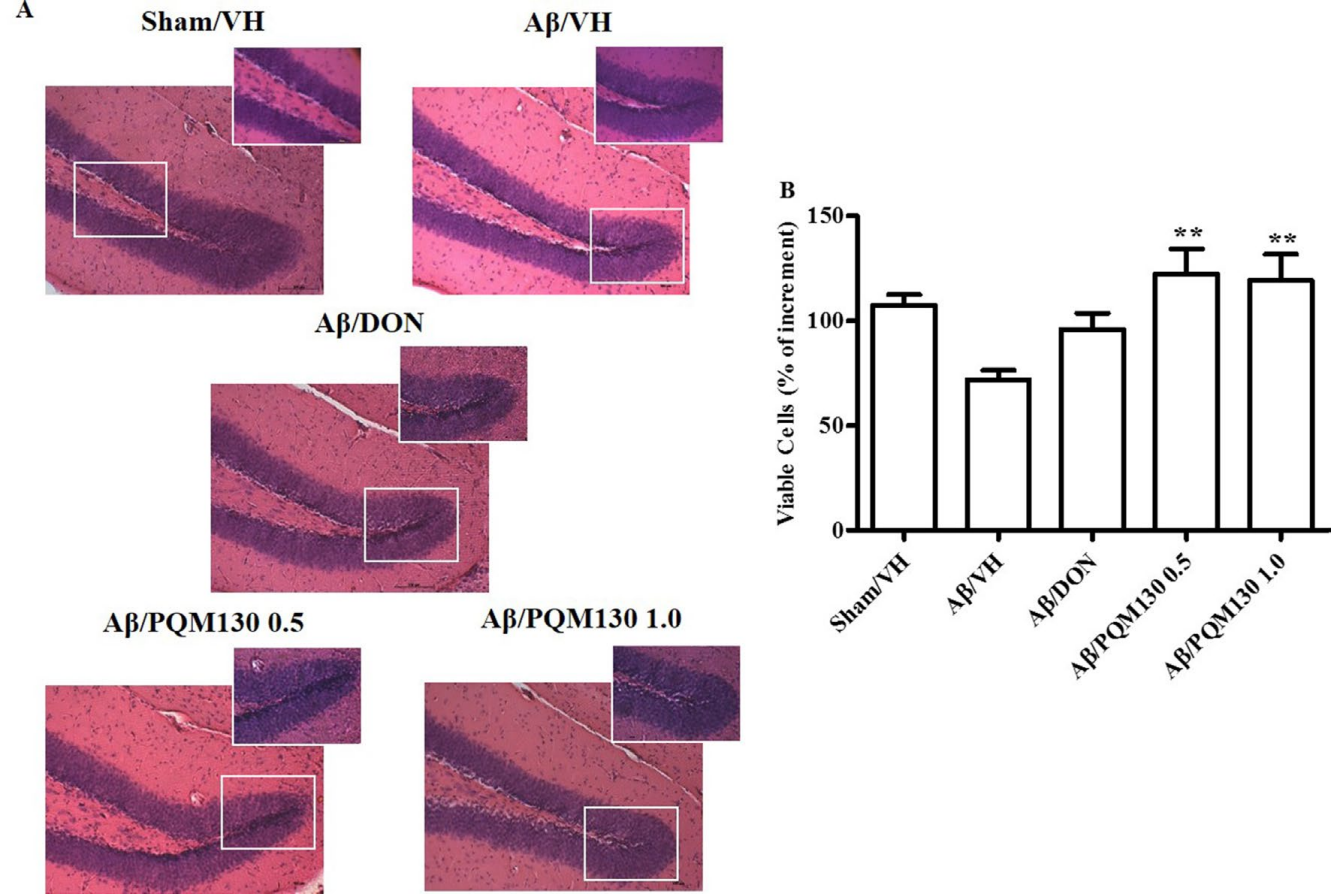
Effects of donepezil and PQM130 treatments (0.5 or 1.0 mg/kg) on neuronal cell death in the Aβ_1-42_O-injected mice. Representative H&E staining of coronal sections containing the hippocampus. Magnification, 20× and 40×; scale bar, 100 µm **(A)**. Quantitative analysis of H&E staining **(B)**. The values are expressed as mean of % of increment ± SEM (*n* = 10) of the density of each experimental group compared to the Sham/VH group (**B**: **p < 0.01 vs. Aβ/VH; ANOVA, *post hoc* test Bonferroni).

**Figure 6:**
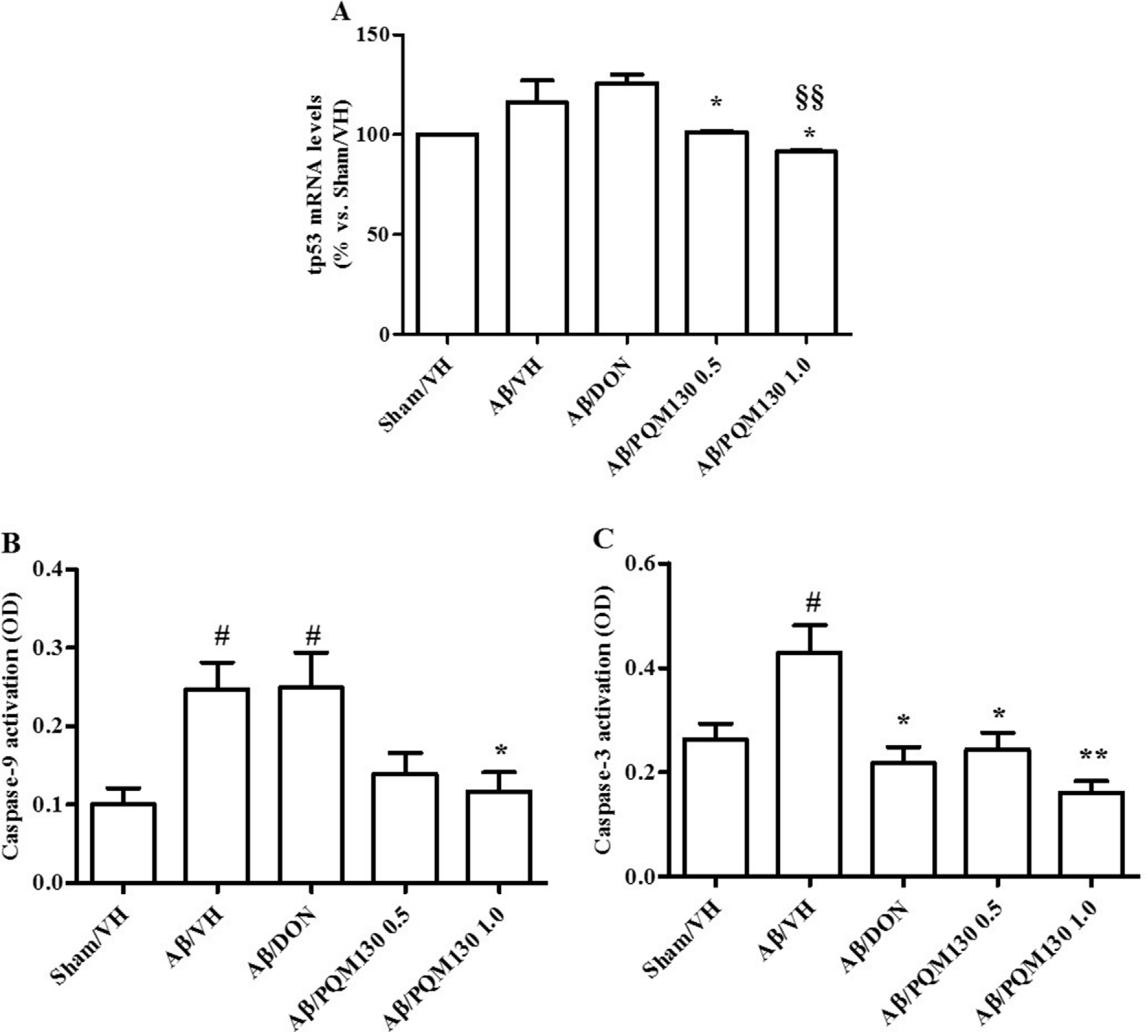
Effects of donepezil and PQM130 treatments (0.5 or 1.0 mg/kg) on tp53 mRNA relative expression **(A)** and caspase-9 **(B)** and caspase-3 **(C)** activations in the Aβ_1-42_O-injected mice. The tp53 mRNA relative expression was determined in hippocampal samples through the 2^−ΔΔCt^ method and represented as percentage vs. the Sham/VH group. ACTB was used as control housekeeping gene. Caspase-9 and -3 activations were determined using a specific chromogenic substrate in the hippocampal samples. The values are expressed as mean ± SEM (*n* = 10) of optical density (OD) of each experimental group (**A**: *p < 0.05 vs. Aβ/VH, ^§§^ *p* < 0.01 vs. Aβ/DON; **B**: ^#^ *p* < 0.05 vs. Sham/VH, *p < 0.05 vs. Aβ/VH; **C**: ^#^ *p* < 0.05 vs. Sham/VH, *p < 0.05 and **p < 0.01 vs. Aβ/VH; ANOVA, *post hoc* test Bonferroni).

We next observed the pathologic changes in different hippocampal areas through H&E-stained sections from the sham, the Aβ/VH group, and the mice under different treatments (DON and PQM130 treatment groups: 1 and 0.5  mg/kg). In the Aβ/VH mice, H&E staining exhibited irregular and sparse neuronal arrangements in the CA1, CA3, and DG regions of the hippocampus. We also observed many unhealthy neurons (**Figure 5A**). Interestingly, PQM130 treatment but not donepezil ameliorated neuronal injury compared with the saline-treated Aβ group (*p* < 0.01, **Figure 5B**). In AD, increased p53 level was detected in various parts of patient brains (Cenini et al., 2008) when compared to the brains of healthy individuals. Likewise, data from animal AD models showed an increase in p53 level in affected neurons (Ohyagi et al., 2005). As could be expected, the Aβ treatment induced the up-regulation of p53 at gene level. On the contrary, PQM130 but not donepezil significantly down-regulated p53 expression (*p* < 0.05, **Figure 6A**). Subsequently, to elucidate the underlying mechanisms of the PQM130 improvement on Aβ-induced neuronal damage, the activations of caspase-9 and -3 were detected. Once activated, the caspase-9 cleaves and activates the effector procaspase-3 triggering the apoptotic pathway. As shown in **Figure 6B and C**, the caspase-9 and -3 were markedly activated in the hippocampal samples of the Aβ_1-42_O-treated group, when compared to the sham group (*p* < 0.05). However, PQM130 treatment was able to inhibit the activation of both caspases induced by Aβ_1-42_O, especially at the highest dose (*p* < 0.05 and *p* < 0.01, respectively), while donepezil was effective to counteract the activation of the caspase-3 but not caspase-9 (*p* < 0.05).

### PQM130 Antagonized Aβ_1-42_O-Induced Oxidative Stress in Mice

**Figure 7:**
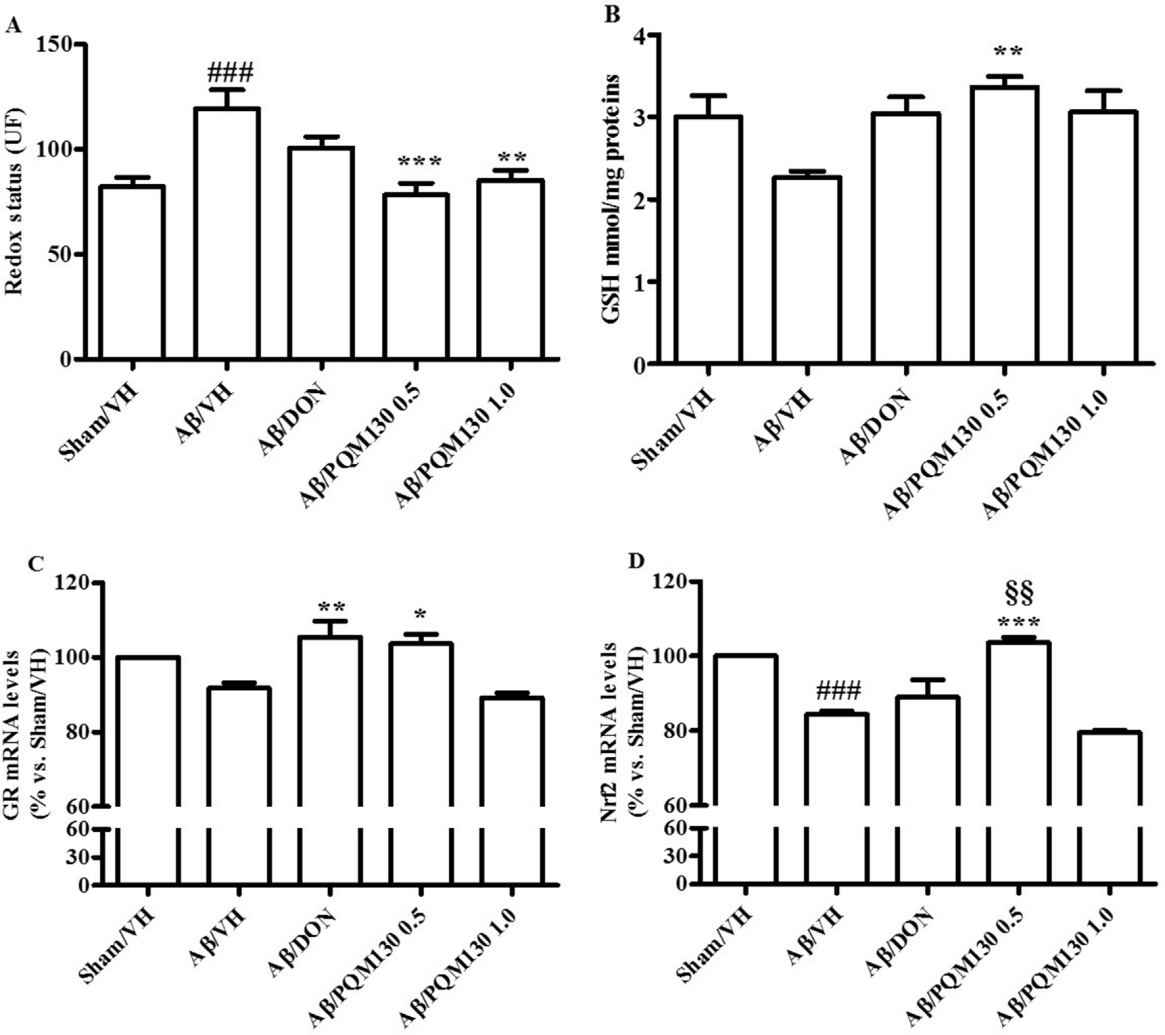
Effects of donepezil and PQM130 treatments (0.5 or 1.0 mg/kg) on cellular redox status in the Aβ_1-42_O-injected mice. Redox status was evaluated in the hippocampal samples based on DCF’s fluorescence emission at 535 nm after excitation at 485 nm. The values are expressed as mean ± SEM (*n* = 10) of fluorescence intensity arbitrary units (UF) of each experimental group **(A)**. GSH content was measured using a colorimetric assay in the hippocampal samples. The values are calculated using a standard calibration curve and expressed as mean ± SEM (*n* = 10) of mmol GSH/mg protein **(B)**. GR and Nrf2 mRNA relative expressions **(C** and **D)** were determined through the 2^−ΔΔCt^ method and presented as percentage vs. the Sham/VH group. ACTB was used as control housekeeping gene. (**A**: ^###^ *p* < 0.001 vs. Sham/VH, **p < 0.01 and ***p < 0.001 vs. Aβ/VH; **B**: **p < 0.01 vs. Aβ/VH group; **C**: *p < 0.05 and **p < 0.01 vs. Aβ/VH group; **D**: ^###^ *p* < 0.001 vs. Sham/VH group, ***p < 0.001 vs. Aβ/VH group, ^§§^ *p* < 0.01 vs. Aβ/DON group; ANOVA, *post hoc* test Bonferroni).

As shown in **Figure 7A and B**, the Aβ_1-42_O injection induced a predictable oxidative stress to the mice brain, as underlined by significant increased ROS formation (*p* < 0.001) and decreased GSH levels in the hippocampal samples compared to the sham group. However, the administration of PQM130, but not donepezil, resulted in the significant decrease of ROS compared with the Aβ/VH group (*p* < 0.001 and *p* < 0.01, respectively). Moreover, PQM130 treatment increased GSH levels in the hippocampi of the Aβ/VH mice close to the sham group levels, particularly with the 0.5 mg/kg dose group (*p* < 0.01). In addition, we carried out gene expression profiling as an effective biomarker to detect cellular stress. In this study, the gene expression analysis for GR enzyme and Nrf2 demonstrated that Aβ treatment decreased GR mRNA expression levels, while donepezil and PQM130 (0.5 mg/kg) significantly increased GR mRNA levels (*p* < 0.01 and *p* < 0.05, respectively; **Figure 7C**). As expected, the expression of Nrf2 was found to be significantly decreased in the hippocampi of the Aβ/VH mice (*p* < 0.001); conversely, PQM130 (1 mg/kg) treatment markedly up-regulated the mRNA levels of Nrf2, compared to the Aβ/VH mice (*p* < 0.001).

### PQM130 Regulated GSK3β and ERK1/2 Protein Expressions in Mice

**Figure 8:**
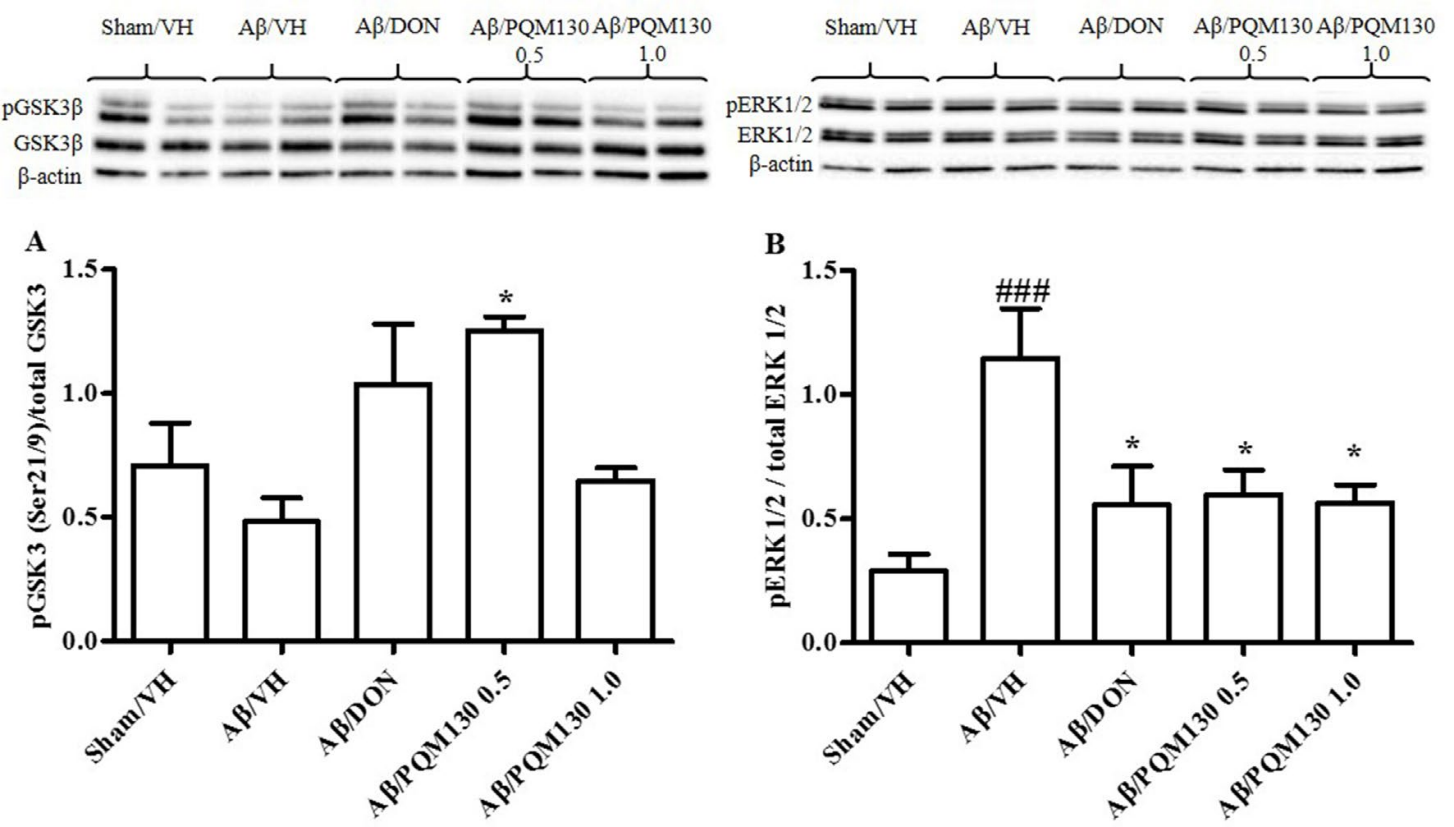
Effects of donepezil and PQM130 treatments (0.5 or 1.0 mg/kg) on GSK3 **(A)** and ERK1/2 **(B)** phosphorylations (pGSK3β Ser21/9 residue and pERK1/2) in the Aβ_1-42_O-injected mice. pGSK3β and pERK1/2 were determined by Western blotting in the hippocampal samples at 46 and 42/44 kDa, respectively, and using total GSK3, total ERK1/2, and β-actin (42 kDa) as loading control. Top: representative images of pGSK3β, GSK3, and β-actin **(A)** and pERK1/2, ERK1/2, and β-actin **(B)** expressions in hippocampus. Bottom: quantitative analysis of the Western blotting results for the pGSK3β **(A)** and pERK1/2 **(B)** levels. The graphs show densitometry analysis of the bands appertaining to the protein of interest. The values are expressed as mean ± SEM (*n* = 10) of each group. (**A**: *p < 0.05 vs. Aβ/VH group; **B**: ^###^ *p* < 0.001 vs. Sham/VH, *p < 0.05 vs. Aβ/VH group; ANOVA, *post hoc* test Bonferroni).

Because glycogen synthase kinase 3β (GSK3β) played a pivotal role in the pathogenesis of AD (Llorens-Martín et al., 2014), we examined the phosphorylation levels of GSK3β (Ser9) to investigate its potential involvement in the PQM130 mechanism of neuroprotection (**Figure 8A**). As shown in **Figure 8A**, the levels of phosphorylated GSK3β was decreased, although not significantly, in the Aβ/VH group. However, the treatment with PQM130 at a dose of 0.5 mg/kg significantly increased the levels of phosphorylated GSK3β protein (*p* < 0.05). In addition, the phosphorylation of ERK1/2 was also detected in our model, since the MAPK/ERK1/2 signaling pathway is involved in the modulation of neuronal apoptosis and may contribute to AD pathogenesis (Morroni et al., 2016). Results found the Aβ_1-42_O injection increased the phosphorylation of ERK1/2 compared with the sham group (*p* < 0.001). However, treatment with PQM130 and donepezil markedly repressed the phosphorylation of ERK1/2 induced by Aβ_1-42_O (*p* < 0.05, **Figure 8B**), indicating that the dephosphorylation of ERK1/2 concurred to the anti-apoptotic effect of PQM130.

### PQM130 Reduced Aβ_1-42_O-Induced Astrocytic Activation in Mice

**Figure 9:**
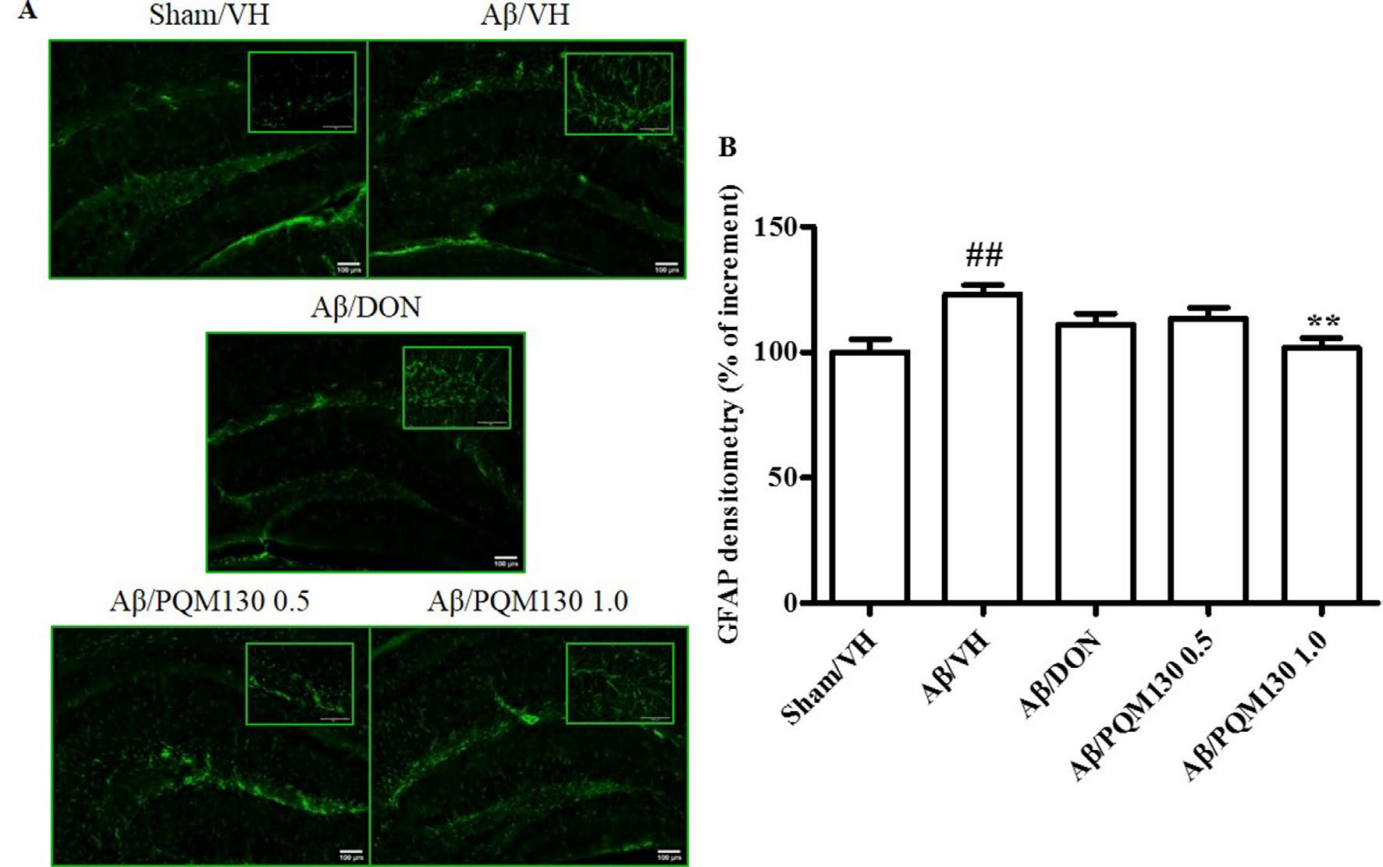
Effects of donepezil and PQM130 treatments (0.5 or 1.0 mg/kg) on astrocyte activation in the Aβ_1-42_O-injected mice. Representative photomicrographs **(A)** of immunostaining for GFAP in brain coronal sections containing hippocampal structure of each experimental group. Magnification, 10× and 40×; scale bar, 100 µm. Quantitative analysis of GFAP immunostaining **(B)**. The values are expressed as mean of % of increment ± SEM (*n* = 10) of the fluorescent intensity of each experimental group compared to the Sham/VH group (**B**: ^##^ *p* < 0.01 vs. Sham/VH, **p < 0.01 vs. Aβ/VH; ANOVA, *post hoc* test Bonferroni).

To examine the effects of PQM130 on neuroinflammation induced by Aβ_1-42_O, we performed immunohistochemical staining for the astrocyte marker GFAP. The quantitative analysis showed that the percentages of the GFAP-stained hippocampal areas were markedly increased in the Aβ/VH group compared with the Sham/VH group (*p* < 0.01). However, in the PQM130-treated mice (1 mg/kg), GFAP-positive areas decreased (*p* < 0.01, **Figure 9B**) compared to those in the vehicle-treated Aβ_1-42_O mice. These results suggested that PQM130 treatment alleviated the neuroinflammation induced by Aβ_1-42_O in the AD brain.

### PQM130 Modulated Synaptic Plasticity in Mice

**Figure 10:**
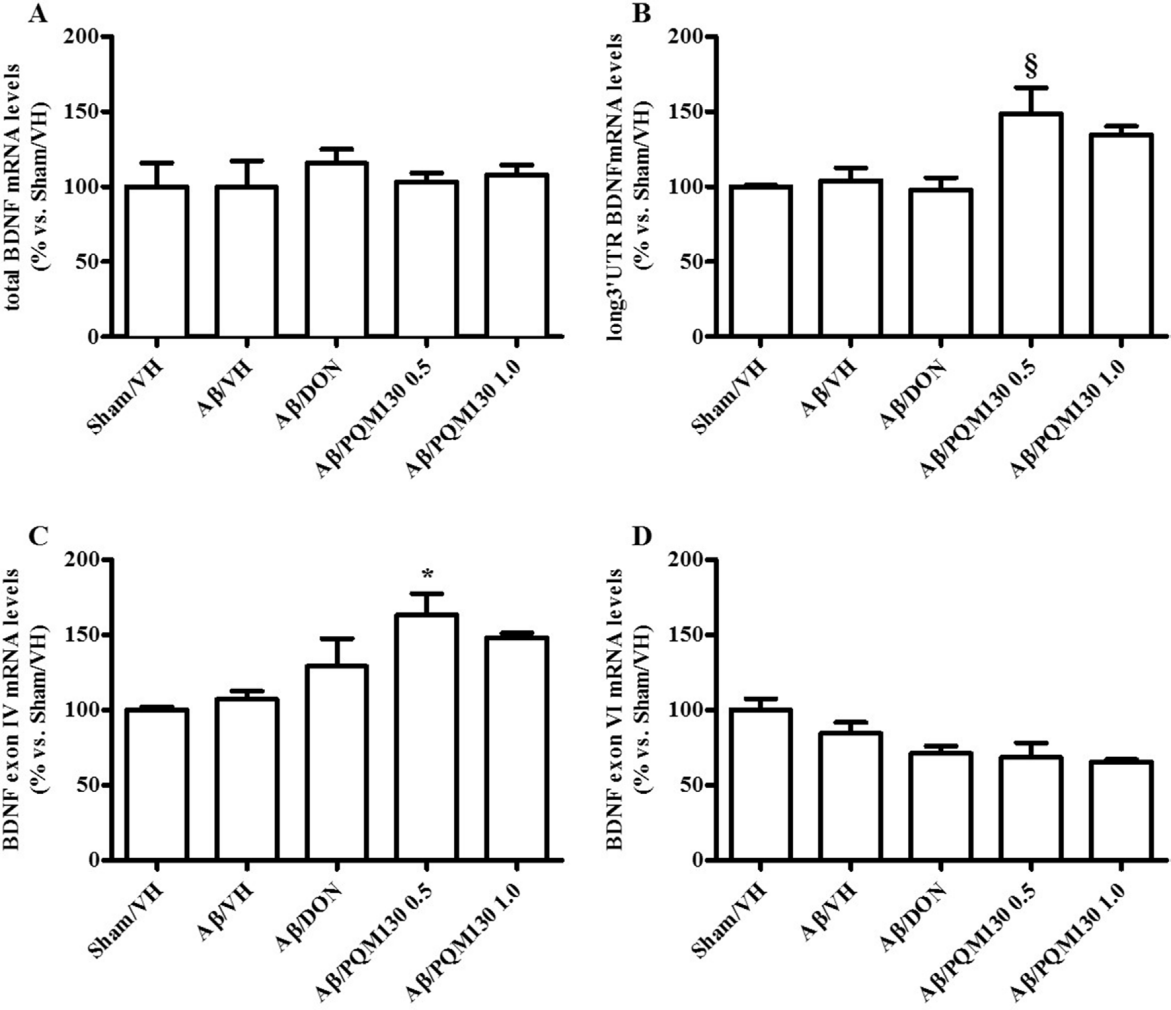
Effects of donepezil and PQM130 treatments (0.5 or 1.0 mg/kg) on the total BDNF **(A)**, long 3′UTR BDNF **(B)**, BDNF exon IV **(C)**, and BDNF exon VI **(D)** mRNA relative expressions in the Aβ_1-42_O-injected mice. The mRNA relative expressions were determined in the hippocampal samples through the 2^−ΔΔCt^ method and represented as percentage vs. the Sham/VH group. ACTB was used as control housekeeping gene. (**B**: ^§^ *p* < 0.05 vs. Aβ/DON; **C**: *p < 0.05 vs. Aβ/VH; ANOVA, *post hoc* test Bonferroni).

**Figure 11:**
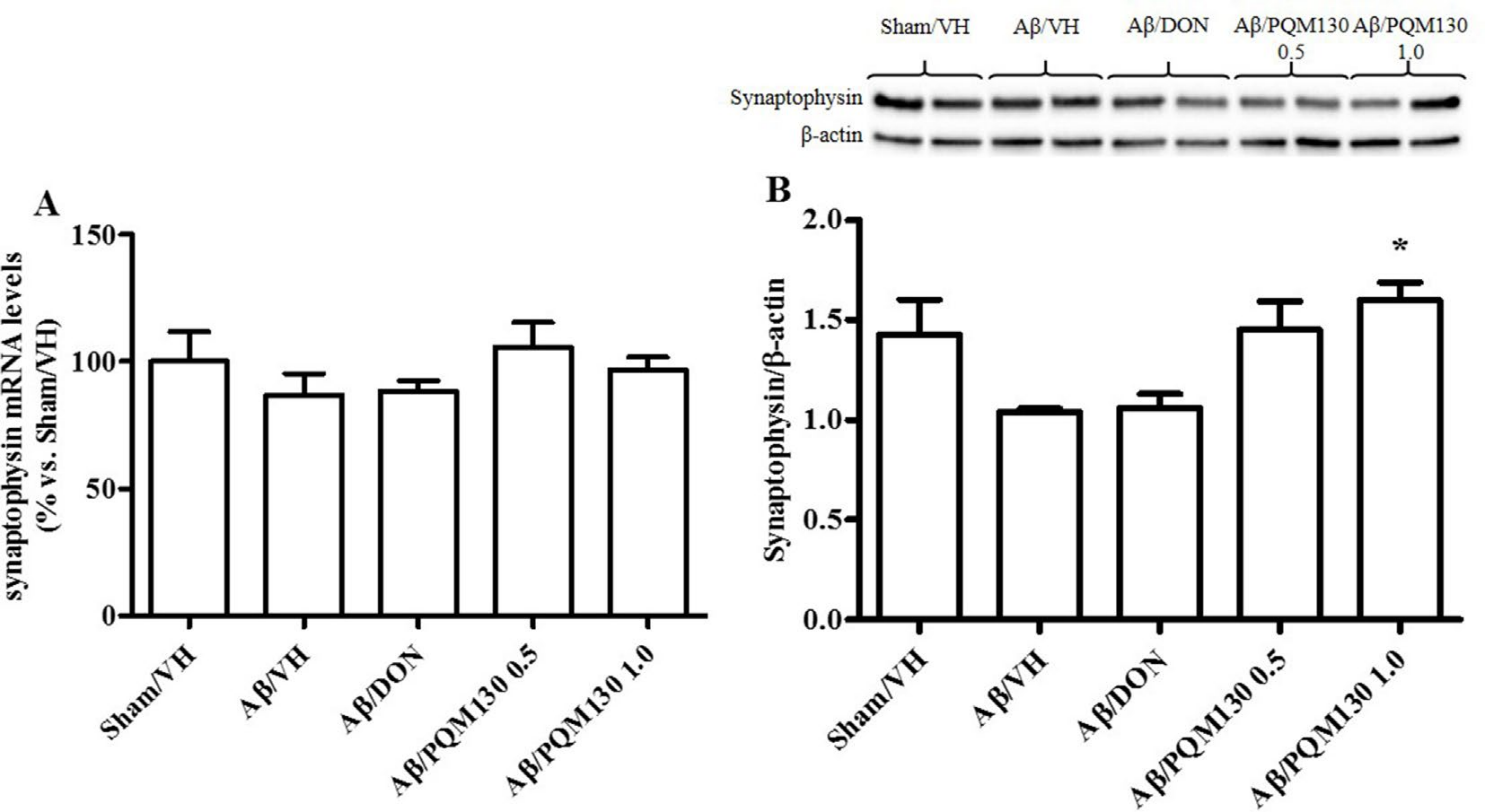
Effects of donepezil and PQM130 treatments (0.5 or 1.0 mg/kg) on sinaptophysin levels in the Aβ_1-42_O-injected mice. Synaptophysin mRNA relative expressions in the hippocampal samples **(A)**. The mRNA relative expressions were determined through the 2^−ΔΔCt^ method and represented as percentage vs. the Sham/VH group. ACTB was used as control housekeeping gene. Synaptophysin activation was determined by Western Blotting in hippocampal samples at 33 kDa using β-actin (42 kDa) as loading control **(B)**. Top: representative images of synaptophysin and β-actin expressions in hippocampus. Bottom: quantitative analysis of the Western blotting results for the synaptophysin levels. The values are expressed as mean ± SEM (*n* = 10) of each experimental group. (**B**: *p < 0.05 vs. Aβ/VH; ANOVA, *post hoc* test Bonferroni).

Firstly, we analyzed the total BDNF gene expression in our samples and the results did not show any significant difference among the different experimental groups (**Figure 10A**). In order to clarify the different responsiveness to PQM130, the expression profile of some neurotrophin transcripts, namely, long 3′UTR BDNF and exons IV and VI, were investigated (**Figure 10B–D**). In deep, PQM130 (1 mg/kg) increased significantly the expression of long 3′UTR BDNF (*p* < 0.05, **Figure 10B**) and isoform IV (*p* < 0.05, **Figure 10C**), whereas no changes were found in the other experimental groups. Classic effects of BDNF consist of promoting differentiation, migration, and dendritic arborization, and enhancing neuronal viability. In addition to these recognized actions, recent findings highlighted that BDNF affects development, function, and plasticity in the synapse (Kuczewski et al., 2009). Thus, we next investigated the effect of PQM130 on the pre-synaptic protein synaptophysin. As shown in **Figure 11A**, there is a slight decrease in synaptophysin mRNA levels in the Aβ/VH and Aβ/DON groups, while the values of the PQM130 groups were maintained at the sham group levels. Even more interesting, the Western blot analysis (**Figure 11B**) revealed a more pronounced reduction of synaptophysin expression in the Aβ/VH and Aβ/DON hippocampal samples. However, after PQM130 treatment (1 mg/kg), the expression of synaptophysin was significantly increased as compared to the Aβ/VH group (*p* < 0.05).

## Discussion

The inhibition of AChE activity is the most realistic approach in the symptomatic treatment of mild to moderately severe AD. Patients are currently treated with AChE inhibitors, and among these, the first-line symptomatic drug is donepezil. In the light of the increasingly accepted conception of AD as a complex pathological network, intensive efforts are being made in the search of new drugs that can simultaneously hit several key biological targets of the network, including AChE. Moreover, AD has decades-long preclinical period (Jack and Holtzman, 2013), which suggests the need to find early therapeutic agents with efficacy at initial stages of the AD pathology. Taking into account all these considerations, the present study aimed to assess the efficacy of the feruloyl–donepezil hybrid PQM130 on AD neurodegenerative processes and on cognitive outcomes, trying to make also a comparison with donepezil activity. In our previous study, PQM130 had already shown an interesting *in vivo* anti-inflammatory activity and *in vitro* metal chelator activity, as well as neuroprotective activity against oxidative damage (Dias et al., 2017). Here, we have elucidated the multifaceted activities of PQM130, like decreasing neuronal death and oxidative stress, improved neurotrophic effect, counteracted inflammation, and ameliorated spatial memory functions as compared to the Aβ_1-42_O lesioned group. It is clear that a successful neuroprotective and neurotrophic strategy could not only delay the progression of neurodegeneration but also provide improvements in the disease condition.

In the MWM test, two main parameters are necessary to locate the hidden platform. Firstly, the mice should develop skills needed to handle the stressful condition, like swimming and recognizing the hidden platform as the only escape route. The second parameter is the spatial learning component, which implies that the mice have to learn exactly the platform’s position and reach it within a minute from the different starting position (Broadbent et al., 2004; Ghumatkar et al., 2015). Here, we found a progressive improvement in the spatial memory as shown by the significant reduction in escape latency time in the PQM130-treated mice as compared to the Aβ/VH mice when evaluated on the 4th and 5th days. This improvement may be ascribed to PQM130’s ability to reduce oxidative stress and AChE activity to finally enhance cholinergic neuronal transmission. The Aβ/DON group showed a swimming performance comparable to the mice treated with the same dose of PQM130. This effect of donepezil may be related to its AChE inhibition (Ghumatkar et al., 2015). However, the probe trial was not implemented significantly in this study, only the time spent in the opposite quadrant markedly decreased after PQM130 and donepezil treatments. Thus, the reduced escape latency time in the PQM130-treated group demonstrates its interesting effect on spatial learning ability. Working memory has been previously reported to be negatively involved in the early stages of AD (Kim et al., 2014; Okamoto et al., 2018), and spontaneous alternation behavior in the Y-maze test may be considered as a reflection of this kind of short-term memory. The continuous spontaneous alternation in the Y-maze test can both elude stressful handling of animals and provide memory and locomotor evaluation (Kirshenbaum et al., 2015). Interestingly, we showed that PQM130 counteracted the negative effect of Aβ_1-42_O on working memory in a dose-dependent manner. This was highlighted by the significant enhancement in percent of alternation behavior in the Y-maze, and the effects of the highest dose of PQM130 are comparable to those of donepezil. Our data are in agreement with previous studies showing that donepezil significantly improves alternation deficits in this test (Meunier et al., 2006; Hu et al., 2012) in the Aβ-injected mice.

Although it is not clearly known how Aβ injection can induce memory impairment in mice, we previously established that Aβ directly caused apoptosis leading to neuronal cell loss and, ultimately, neurodegeneration (Yao et al., 2005; Morroni et al., 2016). The memory and cognitive decline in AD are strongly related to the apoptotic pathway (Obulesu and Lakshmi, 2014; Xu et al., 2017), which involves mitochondrial dysfunction, caspase activation, and DNA fragmentation (Ramalho et al., 2008). Our data showed that the hippocampal damage and caspase-9 and -3 activations in lesioned mice were markedly reversed by PQM130. Meanwhile, donepezil did not show the same effectiveness in counteracting apoptosis and neuronal damage. Moreover, increased p53 level is infallibly detectable in brain areas attained by AD, in the corresponding brain areas of animal models, and in neuronal cells isolated from AD brains (Szybińska and Leśniak, 2017). Interestingly, PQM130 substantially reduced the expression of p53, which corroborated its antiapoptotic activity. It is known that p53 directly binds to and increases the activity of GSK3β while inhibition of nuclear GSK3β attenuated p53-dependent transcription (Watcharasit et al., 2002). The link between p53 and GSK3β (i.e., between p53 and tau phosphorylation) may be more complex; however, in this study, we found that the decrease in p53 expression levels after PQM130 treatment is most likely reflected in a phosphorylation (and thus deactivation) of GSK3β, leading to protection against neuronal death induced by Aβ_1-42_O.

Several studies demonstrated that oxidative stress precedes the rise of senile plaques and neurofibrillary tangles, therefore leading to dementia’s symptoms (Wang et al., 2014; Tian et al., 2019). Indeed, the increase of Aβ and oxidative stress, causing neuronal cell death, are common mechanisms in the progression of AD (Lee et al., 2011). Here, we found that PQM130 and not donepezil significantly ameliorates oxidative damage as demonstrated by the increase of GSH levels and GR and Nrf2 expressions in the Aβ_1-42_O-treated mice, confirming the similar evidences recorded by PQM130 in neuronal SH-SY5Y cells (Dias et al., 2017). The role of Nrf2 in Aβ-induced oxidative stress is controversial (Rong et al., 2018). Ramsey and colleagues described that in AD brains, Nrf2 is mostly found in the cytoplasm in its inactive form, which means that Nrf2 does not trigger the expression of antioxidant enzymes (Ramsey et al., 2007). Sarkar and colleagues demonstrated that Aβ_25-35_ increased oxidative stress and suppressed Nrf2 activation (Sarkar et al., 2017). Moreover, Branca et al. showed that reducing Nrf2 levels exacerbated cognitive impairments in a transgenic model of AD. They also speculated that “Nrf2 might act as a molecular link between brain aging and AD” (Branca et al., 2017). Numerous laboratories supported this hypothesis showing that Nrf2 activity decreased with aging (Suh et al., 2004; Zhang et al., 2015; Li et al., 2018). Moreover, Nrf2 activity is strictly related to tau pathology, enhancing the link between Nrf2 and AD (Lastres-Becker et al., 2014). In our model, exposure to Aβ_1-42_O caused a marked decrease in Nrf2 activation, and only PQM130 significantly increased its expression, probably due to the presence of ferulic acid in this hybrid molecule. In this regard, the presence of α, β-unsaturated carbonyl system in PQM130 suggests the ability of this molecule to activate Nrf2 through a Michael addition reaction (de Freitas Silva et al., 2018). Thus, the effect of PQM130 on Aβ_1-42_O-induced oxidative injury could explain the ability of PQM130 to counteract apoptotic cell death and cognitive impairment observed in our model.

Activity of ERK1/2 is modulated by ROS, and several studies demonstrated its activation in different AD models (Zhu et al., 2002; Chong et al., 2006; Gan et al., 2014; Chang et al., 2018). Moreover, inhibition of ROS formation decreased ERK1/2 activation in an AD model (Kim et al., 2009). The ERK pathway is fundamental to memory consolidation and synaptic plasticity in the hippocampus. Moreover, the fine regulation of ERK is crucial for the hippocampal functions (Goedert and Spillantini, 2006). Notably, in our model, Aβ_1-42_O contributed to the abnormal activation of ERK1/2 and there was an obvious decrease of p‐ERK1/2 levels by PQM130 administration.

ERK activation is also found in reactive astrocytes, affecting Aβ production through ROS formation (Kim and Wong, 2009). Therefore, compounds inactivating astrocytes and MAPK pathways could reduce Aβ formation and thus prevent or counteract neuronal injury in the AD brain (Butterfield, 2002; Lee et al., 2011). Additionally, glial cells and their resident protein GFAP are able to combine neuronal input, control synaptic activity, and translate signals tightly linked to learning and memory by the formation of cytoskeletal filaments (Konar et al., 2011; Ghumatkar et al., 2015). Our results showed that PQM130 and not donepezil might alleviate reactive gliosis, by reason of the ability of this treatment to reduce levels of GFAP in the hippocampus of the Aβ_1-42_O-lesioned mice.

BDNF belongs to the neurotrophin family of survival-promoting molecules. It exerts significant protective effects on fundamental neuronal pathways altered in AD (Nagahara et al., 2009). The transcription of the BDNF gene is very elaborated. At least eight promoters encode to different mRNA transcripts, each containing a 5′ exon spliced to a common 3′ coding exon, and all of which generate the same BDNF protein (Aid et al., 2007; Chapman et al., 2012). We examined the expression of total BDNF mRNA, two 5′ exon-specific transcripts (IV and VI), and BDNF mRNA transcripts with a long 3′ untranslated region (3′UTR) in the hippocampal samples. BDNF mRNA transcripts with long 3′UTRs play essential roles in dendritic spine morphology and long-lasting synaptic plasticity (An et al., 2008; Chapman et al., 2012). In our model, the expression of these transcripts was not reduced by Aβ_1-42_O injection; however, PQM130 markedly increased long 3′UTR and exon IV. Intriguingly, the increased level of BDNF in the hippocampus was accompanied by an up-regulated expression of synaptophysin. Therefore, these neurochemical results lead us to assume that PQM130 may activate the BDNF signaling pathway and thus control the expression of its downstream signaling components and the structural proteins associated to synaptic plasticity in the hippocampus, improving cognitive deficits in mice.

In conclusion, the results of this study demonstrated the nootropic, neuroprotective, and neurotrophic activities of the multi-target drug PQM130 in our AD experimental model. The nootropic effect could be related to the inhibition of AChE activity and the modulation of neuronal survival pathways, and consequently ameliorating the spatial memory formation. Neuroprotection might be attributed to its high potential as antioxidant, and to its ability to counteract apoptotic death and inflammation. Neurotrophicity might be ascribed to its increased BDNF and synaptophysin levels in the hippocampus. Compared to the first-line treatment donepezil, PQM130 appears a more attractive multipotent therapeutic molecule. Thus, our research findings prospect PQM130 as a promising candidate to be further investigated in AD therapy.

## Data Availability Statement

The raw data supporting the conclusions of this manuscript will be made available by the authors, without undue reservation, to any qualified researcher.

## Ethics Statement

Procedures on the mice were carried out according to the European Communities Council Directive 2010/63/EU and the current Italian Law on the welfare of the laboratory animal (D.Lgs. n.26/2014). The animal protocol was approved by the Italian Ministry of Health (Authorization No. 291/2017-PR) and by the corresponding committee at the University of Bologna.

## Author Contributions

AT, FM, and PH contributed to the conception and design of the study. KD and CV designed and synthesized the molecule PQM130. LP, AS, and IC performed *in silico* studies. GS and AG performed behavioral, biomolecular, and immunohistochemical analysis. GR, MP, and RM performed gene expression analysis. GS performed the statistical analysis. FM and GS wrote the first draft of the manuscript. LP contributed to data analysis. PH contributed reagents/materials/analysis tools. All the authors contributed to the manuscript revision and read and approved the submitted version.

## Funding

This work was supported by Ministero dell’Istruzione, dell’Università e della Ricerca (MIUR), PRIN 2015 (Prot. 20152HKF3Z and 2015SKN9YT003), and Fondazione del Monte di Bologna e Ravenna.

## Conflict of Interest Statement

The authors declare that the research was conducted in the absence of any commercial or financial relationships that could be construed as a potential conflict of interest.
