# Supplementary material for: PQM130, a Novel Feruloyl–Donepezil Hybrid Compound, Effectively Ameliorates the Cognitive Impairments and Pathology in a Mouse Model of Alzheimer’s Disease
Source: Front Pharmacol. 2019 Jun 12;10:658. doi: 10.3389/fphar.2019.00658 (PMC6581760; doi:10.3389/fphar.2019.00658)
Supplement: Supplementary file 1 [file DataSheet_1.pdf]

Supplementary Material 1

Supplementary Table 1. ***In silico*** **ADME data from QikPro 3.1 software (Schrödinger) for PQM130**

ADME parameter	Description of predicted parameters	PQM130	Reference value*
**Reactive FG**	Number of reactive functional groups; the specific groups are listed in the jobname.out file. The presence of these groups can lead to false positives in HTS assays and to decomposition, reactivity, or toxicity problems in vivo. See Appendix 5 of the QikProp User Manual for a complete list	1	0 – 2
**CNS**	Predicted CNS activity on a –2 (inactive) to +2 (active) scale	1	-2 – +2
**mol_MW**	molecular weight	381.471	130 – 725
**donorHB**	Estimated number of hydrogen bonds that would be donated by the solute to water molecules in an aqueous solution. Values are averages taken over a number of configurations, so they can be non-integer	1	0 – 6
**accptHB**	Estimated number of hydrogen bonds that would be accepted by the solute from water molecules in an aqueous solution. Values are averages taken over a number of configurations, so they can be non-integer	5.5	2.0 – 20.0
**QPlogPo/w**	Predicted octanol/water partition coefficient	4.356	-2 – 6.5
**QPlogS**	Predicted aqueous solubility, log S. S in mol dm–3 is the concentration of the solute in a saturated solution that is in equilibrium with the crystalline solid	-5.118	-6.5 – 0.5
**QPlogHERG**	Predicted IC50 value for blockage of HERG K+ channels	-7.39	< -5
**QPPCaco**	Predicted apparent Caco-2 cell permeability in nm/sec. Caco-2 cells are a model for the gut-blood barrier. QikProp predictions are for non-active transport	275.881	< 25 poor<br>>500 great
**QPlogBB**	Predicted brain/blood partition coefficient	-0.688	-3 – 1.2
**QPlogKhsa**	Prediction of binding to human serum albumin	0.708	-1.5 – 1.5
**Hum Oral Absorp**	Predicted qualitative human oral absorption	3	1: low<br>2: medium<br>3: high
**% Hum Oral Absorp**	Predicted human oral absorption on 0 to 100% scale	96.134	>80%: high<br><25%: poor
**PSA**	Van der Waals surface area of polar nitrogen and oxygen atoms.	70.453	7.0 – 200.0
**Rule of Five**	Number of violations of Lipinski's rule of five. The rules are: mol_MW < 500, QPlogPo/w < 5, donorHB ≤ 5, accptHB ≤ 10. Compunds that satisfy these rules are considered drug-like.	0	Max 4
**Rule of Three**	Number of violations of Jorgensen's rule of three. The three rules are: , QPlogS > -5.7, QPPCaco > 22 nm/s, # Primary Metabolites < 7. Compounds with fewer (and preferably no) violations of these rules are more likely to be orally available.	0	Max 3

*gap or recommended value for 95% of known drugs (source: QikProp 3.1 Manual User - Schrödinger Software)
