# Supplementary material for: PQM130, a Novel Feruloyl–Donepezil Hybrid Compound, Effectively Ameliorates the Cognitive Impairments and Pathology in a Mouse Model of Alzheimer’s Disease
Source: Front Pharmacol. 2019 Jun 12;10:658. doi: 10.3389/fphar.2019.00658 (PMC6581760; doi:10.3389/fphar.2019.00658)
Supplement: Supplementary file 2 [file DataSheet_2.pdf]

VEGA QSAR MODELS

Report

Prediction and Applicability Domain analysis for models:

Carcinogenicity model (IRFMN/Antares) 1.0.0

Core version: 1.2.4

1. Prediction Summary

Prediction for compound Molecule 0

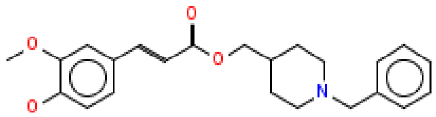

Prediction: Reliability:

Prediction is Possible NON-Carcinogen, the result appears reliable. Anyhow, you should check it through the evaluation of the information given in the following sections.

Compound: Molecule 0
Compound SMILES: O=C(OCC2CCN(Cc1ccccc1)CC2)C=Cc3ccc(O)c(OC)c3
Experimental value: -
Predicted Mutagen activity: Possible NON-Carcinogen
No. alerts for carcinogenicity: 0
Structural alerts: -
Reliability: the predicted compound is into the Applicability Domain of the model
Remarks:
  none

3.1 Applicability Domain:
Similar Compounds, with Predicted and Experimental Values

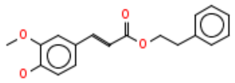

Compound #1

CAS: N.A.
Dataset id: 660 (Training set)
SMILES: O=C(OCCc1ccccc1)C=Cc2ccc(O)c(OC)c2
Similarity: 0.878

Experimental value: NON-Carcinogen
Predicted value: Possible NON-Carcinogen

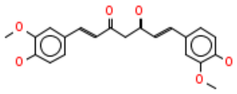

Compound #2

CAS: N.A.
Dataset id: 786 (Training set)
SMILES: O=C(C=Cc1ccc(O)c(OC)c1)CC(=O)C=Cc2ccc(O)c(OC)c2
Similarity: 0.831

Experimental value: NON-Carcinogen
Predicted value: Possible NON-Carcinogen

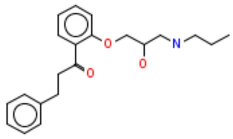

Compound #3

CAS: N.A.
Dataset id: 869 (Training set)
SMILES: O=C(c1ccccc1(OCC(O)CNCCC))CCc2ccccc2
Similarity: 0.792

Experimental value: NON-Carcinogen
Predicted value: Possible NON-Carcinogen

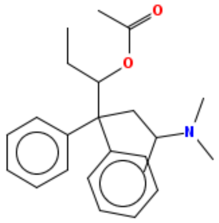

Compound #4

CAS: N.A.
Dataset id: 1450 (Training set)
SMILES: O=C(OC(CC)C(c1ccccc1)(c2ccccc2)CC(N(C)C)C)C
Similarity: 0.792

Experimental value: NON-Carcinogen
Predicted value: Possible NON-Carcinogen

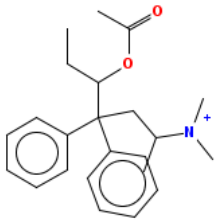

Compound #5

CAS: N.A.
Dataset id: 1226 (Training set)
SMILES: O=C(OC(CC)C(c1ccccc1)(c2ccccc2)CC(C)[NH+](C)C)C
Similarity: 0.791

Experimental value: Carcinogen
Predicted value: Possible NON-Carcinogen

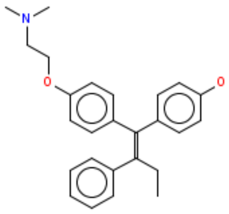

Compound #6

CAS: N.A.
Dataset id: 999 (Training set)
SMILES: Oc1ccc(cc1)C(c2ccc(OCCN(C)C)cc2)=C(c3ccccc3)CC
Similarity: 0.785

Experimental value: NON-Carcinogen
Predicted value: Carcinogen

Alerts (not found in the target): Carcinogenity alert no. 89

3.2 Applicability Domain:
Measured Applicability Domain Scores

Global AD Index
AD index = 0.91
Explanation: the predicted compound is into the Applicability Domain of the model.

Similar molecules with known experimental value
Similarity index = 0.828
Explanation: strongly similar compounds with known experimental value in the training set have been found.

Accuracy of prediction for similar molecules
Accuracy index = 1
Explanation: accuracy of prediction for similar molecules found in the training set is good.

Concordance for similar molecules
Concordance index = 1
Explanation: similar molecules found in the training set have experimental values that agree with the predicted value.

Atom Centered Fragments similarity check
ACF index = 1
Explanation: all atom centered fragment of the compound have been found in the compounds of the training set.

Symbols explanation:

The feature has a good assessment, model is reliable regarding this aspect.

The feature has a non optimal assessment, this aspect should be reviewed by an expert.

The feature has a bad assessment, model is not reliable regarding this aspect.

References and Documentation

You can find complete details on each model and on how to read results in the proper model's guide, available on-line at www.vega-qsar.eu or directly in the VegaNIC application.

Carcinogenicity model (IRFMN/Antares) (version 1.0.0)

QSAR classification model for Carcinogenicity based on a set of rules built with SarPy software extracted from the Antares dataset. Developed by Istituto Mario Negri, Italy; SarPy software developed by Politecnico di Milano, Italy. Model developed inside the VEGA platform.
