# Supplementary material for: PQM130, a Novel Feruloyl–Donepezil Hybrid Compound, Effectively Ameliorates the Cognitive Impairments and Pathology in a Mouse Model of Alzheimer’s Disease
Source: Front Pharmacol. 2019 Jun 12;10:658. doi: 10.3389/fphar.2019.00658 (PMC6581760; doi:10.3389/fphar.2019.00658)
Supplement: Supplementary file 3 [file DataSheet_3.pdf]

VEGA QSAR MODELS

Report

Prediction and Applicability Domain analysis for models:

Mutagenicity (Ames test) CONSENSUS model 1.0.2

Core version: 1.2.4

1. Prediction Summary

Prediction for compound Molecule 0

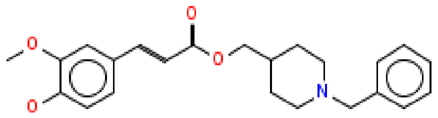

Prediction:

Prediction is NON-Mutagenic with a consensus score of 0.57, based on 4 models.

Compound: Molecule 0
Compound SMILES: O=C(OCC2CCN(Cc1ccccc1)CC2)C=Cc3ccc(O)c(OC)c3
Used models: 4
Predicted Consensus Mutagen activity: NON-Mutagenic
Mutagenic Score: 0
Non-Mutagenic Score: 0.57
Model Caesar assessment: NON-Mutagenic (moderate reliability)
Model ISS assessment: NON-Mutagenic (low reliability)
Model SarPy assessment: NON-Mutagenic (moderate reliability)
Model KNN assessment: NON-Mutagen (good reliability)
Remarks:
none

References and Documentation

You can find complete details on each model and on how to read results in the proper model's guide, available on-line at www.vega-qsar.eu or directly in the VegaNIC application.

Mutagenicity (Ames test) CONSENSUS model (version 1.0.2)

Mutagenicity (Ames test) Consensus model, based on the predictions of the available VEGA mutagenicity models (Caesar, SarPy, ISS and KNN).
